# Effect of Current Density on the Microstructure and Mechanical Properties of 3YSZ/Al_2_O_3_ Composites by Flash Sintering

**DOI:** 10.3390/ma15093110

**Published:** 2022-04-25

**Authors:** Yujie Bai, Ying Zhang, Zhifeng Tian, Junzhan Zhang, Zongmo Shi

**Affiliations:** 1 College of Materials Science and Engineering, Xi’an University of Architecture and Technology, Xi’an 710055, China; baiyj1997@163.com (Y.B.); txh1416524133@163.com (Z.T.); xajzzhang@126.com (J.Z.); shixin2610@163.com (Z.S.); 2 Shaanxi Key Laboratory of Nano Materials and Technology, Xi’an 710055, China

**Keywords:** flash sintering, eutectic structure, grain size, 3YSZ/Al_2_O_3_

## Abstract

The 3YSZ/40 wt% Al_2_O_3_ composites were prepared by flash sintering at a low furnace temperature (700 °C). The effects of the current density on the relative density and Vickers hardness of the composites were systematically investigated. The results showed that the relative densities and Vickers hardness of the samples increased gradually with the increasing of the current densities, and the relative density was as high as 94.2%. The Vickers hardness of 11.3 GPa was obtained under a current density of 102 mA/mm^2^. Joule heating and defects generation are suggested to be the main causes of rapid densification in flash sintering. The microstructure of the molten zone showed the formation of eutectic structures in the composite, suggesting that grain boundary overheating may have contributed to the formation of the molten zone.

## 1. Introduction

Flash sintering is an innovative sintering technique that was first developed by Raj in 2010, initially demonstrated for 8 mol% yttria-stabilized cubic zirconia (8YSZ) [1]. Compared with the conventional sintering method, flash sintering allows for a sintering process at a low furnace temperature in seconds, thus saving sintering costs [2]. Until now, flash sintering has been applied to a wide range of material systems including ionic conductors such as rare earth doped cerium dioxides (SDC, GDC [3,4]), semiconductors (ZnO [5]), insulators (Al_2_O_3_ [6]) and electronic conductors (Co_2_MnO_4_ [7]). However, most of the studies in the field of flash sintering have focused on single-phase ceramic materials, while there have been relatively few reports on the preparation of composites with excellent properties by flash sintering.

The combination of the high toughness of yttrium-stabilized zirconia (YSZ) and the high hardness of alumina (Al_2_O_3_) can effectively bring out their respective advantages, making the composite materials widely used in industrial, aerospace, and defense applications [8,9,10]. However, the preparation of a YSZ/Al_2_O_3_ composite by conventional sintering requires high temperatures (≥1400 °C) and a long sintering time. Flash sintering presents a new opportunity for the rapid and efficient preparation of a high-performance multiphase YSZ/Al_2_O_3_ composite. YSZ/Al_2_O_3_ composites are one of the most studied multiphase materials for flash sintering, and they are excellent model systems combining ionic conductors and insulators. Since the addition of alumina to zirconia can increase the grain boundary conductivity, this greatly improves the densification rate of the YSZ/Al_2_O_3_ composite [11,12,13]. Naik et al. [14] first investigated 50 vol.% YSZ/Al_2_O_3_ composites, mainly including flash sintering initiation conditions and grain coarsening. Recently, Ojaimi et al. [15] compared the best sample obtained by flash sintering with a conventionally sintered sample and found that the grain size was comparable to that obtained by conventional sintering, but while using a much lower furnace temperature than in conventional sintering. To date, most works have typically explored the flash sintering temperature and the effect of flash sintering conditions (electric field and current density) on the microstructural characteristics of a composite under a constant heating rate, whereas the preparation of a composite by flash sintering under a constant furnace temperature has not been systematically investigated.

In this work, 3YSZ/Al_2_O_3_ composites were prepared by flash sintering at different current densities under a constant furnace temperature. The results of the grain size, relative density, and Vickers hardness of flash-sintered composites were compared with conventional sintered composites, and the mechanism of flash sintering was discussed. In addition, the molten zone was found and analyzed in the microstructure of the composites at a current density of 102 mA/mm^2^.

## 2. Experimental Section

The 3YSZ/Al_2_O_3_ composites were prepared by commercial powders of 3 mol% Y_2_O_3_ stabilized ZrO_2_ (average particle size: 40 nm; indicated to be ≥99.8%) from Nanjing Emperor Company and α-alumina (AKP-53, average particle size: 180 nm; indicated to be ≥99.99%) from Sumitomo Chemical Company. The 3YSZ and Al_2_O_3_ powders, with a mass fraction ratio of 60/40, were mixed in anhydrous ethanol by ball milling for 6 h. After drying and grounding, the powders were mixed uniformly with a 5 wt% aqueous solution of PVA and were then uniaxially pressed into a cylindrical platelet (diameter of 10.0 mm and height of 6.0 ± 0.2 mm) under 370 MPa. The platelets were pre-sintered at 1000 °C for 2 h, and then silver was painted on the top and bottom surfaces of the sample to make good contact with the electrodes.

For the flash sintering experiment, a constant electric field *E* = 400 V/cm was applied after holding at 700 °C for 10 min. Different current densities of *J* = 51, 64, 76, 89, 102 mA/mm^2^ were used, the holding time was fixed to 120 s, and the samples were noted as FS-51, FS-64, FS-76, FS-89, FS-102, respectively. The electric field was provided by a DC power supply (DCPS5023, Lanyi, Hangzhou, China), and the recorded data of current and voltage were collected using digital multimeters (VC86E, Victor, Shenzhen, China). A displacement sensor was placed in the upper plane of the furnace to monitor the volume change in the vertical direction of the sample. In addition, the sample was also prepared by conventional sintering at 1400 °C for 2 h to compare both processes.

The crystalline phases of all the samples were analyzed by X-ray diffraction (XRD, DMAX U1TIMA IV, Rigaku, Tokio, Japan) with Cu Kα (10° ≤ 2θ ≤ 90°). The polished cross sections were observed by Scanning Electron Microscopy (SEM, GEMINI SEM 500, Zeiss, Jena, Germany) after they had been thermally etched at 1350 °C for 15 min. The grain sizes of the composites were measured by the statistical software Nano Measurer. The relative densities of the composites were measured by Archimedes’ method. The hardness of composites was characterized by the micro-Vickers hardness tester (HXD-2000TMC/LCD, Taiming, Shanghai, China). A Vicker diamond pyramid indenter was pressed into the surface of the sample at a peak load of 19.6 N and holding time of 15 s.

## 3. Results and Discussion

**Figure 1:**
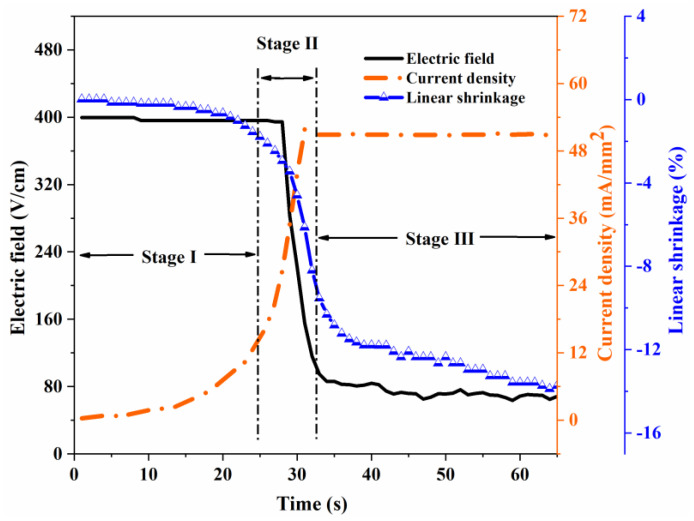
Electric field, current density and linear shrinkage as a function of time during flash sintering.

The typical behavior of the electric field, current density, and the linear shrinkage as a function of time at the current density of 51 mA/mm^2^ are shown in Figure 1. It can be seen distinctly that the flash sintering process was divided into three stages, namely, the incubation stage (Stage I), the transient stage including the current density increasing abruptly, the voltage decreasing and the onset of the flashover (Stage II), and the quasi-steady state regime under current control (Stage III). A sharp shrinkage of the sample was observed in Stage II, which was the main stage of sintering densification, and the sample continued to be sintered in Stage III but shrank to a lesser extent.

**Figure 2:**
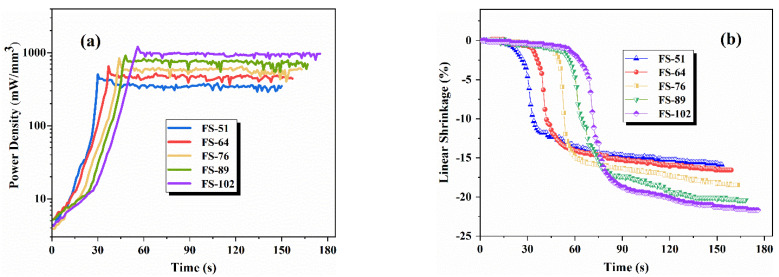
The dependence of (**a**) power dissipation and (**b**) linear shrinkage on time under a constant electric field of 400 V/cm.

Figure 2 shows the sintering dynamics of the 3YSZ/Al_2_O_3_ composites after the input of the electric field at different current densities during flash sintering. Figure 2a shows the fluctuation of power dissipation at different current densities. The power density (*P*) was calculated according to the product of *E* and *J*. The transition from the control mode of voltage to the current created a spike of power dissipation in stage II. Both the peak of power dissipation and power dissipation at the steady state increased with the increase of the current density. For current densities of 51, 64, 76, 89, and 102 mA/mm^2^, the peak values of power dissipation were 507, 652, 835, 914, and 1206 mW/mm^3^, respectively. In stage III, the power dissipations were reduced to about 357, 468, 579, 741, and 895 mW/mm^3^, respectively.

Figure 2b shows the linear shrinkage behavior of the 3YSZ/Al_2_O_3_ composites during flash sintering at different current densities. The shrinkage of the sample increased slowly at the beginning of flash sintering with the increase of the current density because of the gradual increase of the incubation time. The time for the sample to shrink violently increased with the current limit due to the same rate of current increase in the experiment. However, the overall sintering time was still within a very narrow range. As shown earlier, the instantaneous shrinkage in Stage II represented the rapid densification of the sample. The results suggested that the total linear shrinkage of the composites increased from 16.6% to 21.7% when the current density increased from 51 mA/mm^2^ to 102 mA/mm^2^.

**Figure 3:**
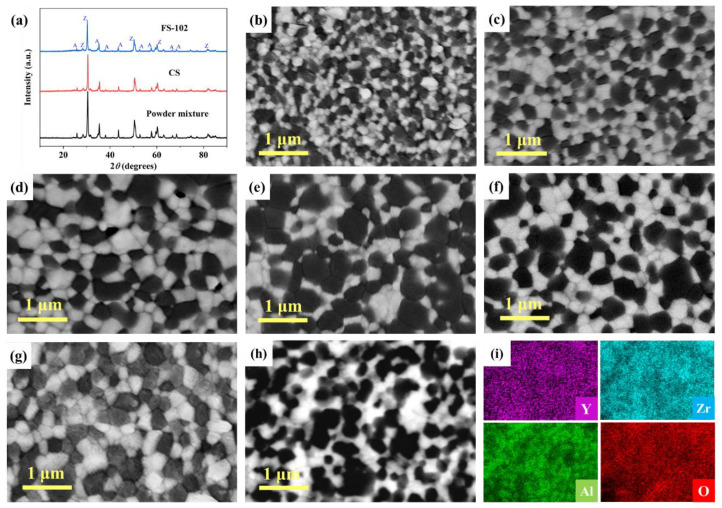
(**a**) XRD patterns of FS-102, conventional sintered sample (CS) and starting powder mixture. SEM images of 3YSZ/Al_2_O_3_ composites flash-sintered at the different current densities (**b**) FS-51, (**c**) FS-64, (**d**) FS-76, (**e**) FS-89 and (**f**) FS-102 and of the (**g**) conventional sintered 3YSZ/Al_2_O_3_ composite. (**h**) SEM image and (**i**) EDS maps of FS-102.

Figure 3a shows the XRD patterns of the samples. Both the flash-sintered sample (FS-102) and conventional sintered sample (CS) were only composed of ZrO_2_ phase (Z) and Al_2_O_3_ phase (A), indicating that the electric field applied at both ends of the samples did not produce any additional phases. Figure 3b–g shows the microstructure of the 3YSZ/Al_2_O_3_ composites flash-sintered at different current densities and of the conventional sintered 3YSZ/Al_2_O_3_ composite. The SEM image and EDS maps of FS-102 are shown in Figure 3h–i. It can be seen that the elements are uniformly distributed. From the distribution maps of Al and Zr elements, it could be determined that the darker regions corresponded to alumina and that the brighter regions were zirconia. The Al_2_O_3_ and ZrO_2_ grains were well dispersed without any aggregation.

As can be seen from Table 1, the average grain sizes of ZrO_2_ increased from 0.18 μm to 0.33 μm, and the average grain sizes of Al_2_O_3_ increased from 0.22 μm to 0.47 μm when the current density increased from 51 mA/mm^2^ to 102 mA/mm^2^. It can also be found that the relative density was less than 85.5% when the current density was below 76 mA/mm^2^, and that it reached a maximum value of 94.2% at a current density of 102 mA/mm^2^. This finding was consistent with Jia [16], indicating that the current density presented a significant effect on the densification and grain size of the 3YSZ/Al_2_O_3_ composite. Moreover, the relative density of the FS-102 sample was found to be higher than that of the sample obtained by conventional sintering (93.2%). However, the furnace temperature was only 700 °C during the flash sintering experiment, which was not sufficient to obtain a high relative density for the 3YSZ/Al_2_O_3_ composite, indicating that the sample temperature reached the high threshold.

Generally, a balance was reached between power dissipation and blackbody radiation loss in Stage III during the flash sintering experiment. Therefore, the sample temperature *T* could be estimated by the blackbody radiation Equation (1) [17]:

(1) $$\;\frac{T}{T_{0}}=\alpha {\left[1+\frac{1000P}{\sigma T_{0}^{4}}\left(\frac{V}{A}\right)\right]}^{1/4}$$

where *T*_0_ is the furnace temperature, *P* is the power dissipation at the steady state, *V*/*A* is the volume-to-surface ratio of the sample, *α* is the emissivity, which is assumed to be 0.8, and *σ* is Stefan’s constant, which is 5.67 × 10^−8^ W·m^2^·K^−4^. It was observed from Table 1 that the estimated temperatures of the samples were 1130, 1221, 1297, 1391, and 1467 °C at current densities of 51, 64, 76, 89, and 102 mA/mm^2^, respectively. Compared with conventional sintering, the sample reached a high temperature of 1467 °C at a current density of 102 mA/mm^2^, but this temperature was not sufficient to densify the sample within 120 s. This is probably because the predicted temperature was the sample temperature in Stage III, while the actual temperature of the sample was higher than the value under the transient stage of flash sintering [18].

Besides the Joule heat mechanism, the generation of defects is considered to be another mechanism that explains the rapid densification of flash sintering [19,20,21]. A high density of charged defects (interstitials and vacancies) was formed on the grain surface/contact points under the combination of the electric field and temperature when the flash event occurred, which dramatically raised the sintering rate and could explain the unusually rapid densification discussed above. Whether it was a high density of charged defects or contacts at the grain surfaces, the complete densification of the samples required a constant current for a few seconds (Stage III). This last stage may have a substantial effect on the enhancement of grain growth or the coarsening of the pristine nanograins [22,23]. Therefore, the average grain size of the FS-102 sample was larger than that of the CS sample from Table 1. Consequently, it is concluded that Joule heating cannot be solely responsible for the densification of flash sintering.

**Figure 4:**
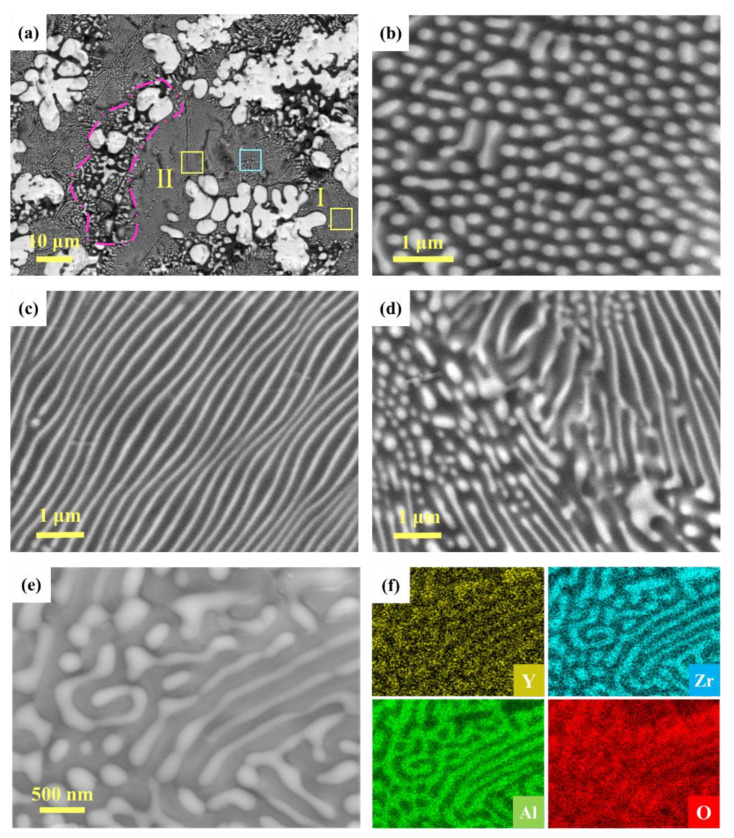
(**a**) SEM image of the molten zone of FS-102 (see the area surrounded by the pink line for colony structure). (**b**,**c**) The microstructure of rod and lamellae structures (see yellow rectangle I and yellow rectangle II in (**a**)). (**d**) Enlargement of the area in the blue rectangle in (**a**). (**e**) SEM image and (**f**) EDS maps of the molten zone of FS-102.

Further observation of the microstructure of the sample revealed the presence of a non-molten zone and molten zone when the current density was 102 mA/mm^2^. The non-molten zone consists of a uniform distribution of Al_2_O_3_ and ZrO_2_ grains, as shown in Figure 3f. The molten zone was close to the anode of the FS-102 sample and accounted for about one-third of the sample thickness. The overall low magnification morphology of the molten zone is shown in Figure 4a. Three different types of structures can be seen in the molten zone, including colony (see the area surrounded by the pink line), regular (see the yellow rectangles), and irregular structures (see the blue rectangle). The regular structures including rod or lamellae are shown in Figure 4b−c, and the irregular structures are shown in Figure 4d. Figure 4e–f shows the SEM image and EDS maps of the molten zone of FS-102. It can be seen that the elements are uniformly distributed, which is consistent with the results in the non-melting zone. This further indicates that the melting of the sample did not cause a local enrichment of the elements.

The macroscopic thermal gradient caused by heat loss at the electrode reached the melting point of the ZrO_2_/Al_2_O_3_ composite (about 1860 °C) [24,25] at some points, resulting in the formation of hot spots inside the sample, which caused a partial melting of the sample. In terms of electrical conductivity, the current through the zirconia grains was much larger than that through the alumina grains, and the increase in the zirconia grains’ boundary temperature led to the rapid wetting of the surrounding alumina grains [26,27]. Therefore, the preferential growth of ZrO_2_ grains resulted in the formation of the coarse ZrO_2_ phase of the colony structures. The regular structures were generated near the boundary of the colony structures, and the irregular structures were uniformly distributed around regular structures. These morphologies were similar to the results reported in relevant papers [15,28] and were considered to be Al_2_O_3_/ZrO_2_ eutectic structures. As a result, it is necessary to consider the possibility that the local liquid phase generated during flash sintering is due to grain boundary overheating [15].

**Figure 5:**
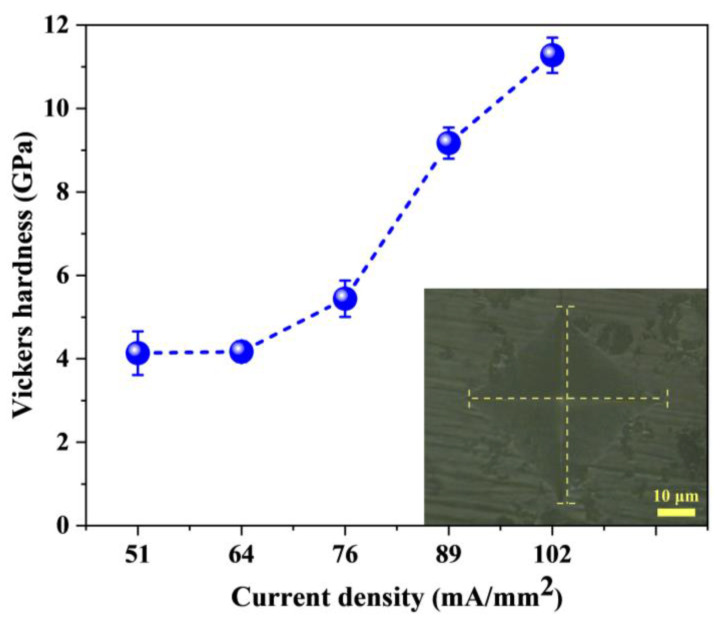
Influence of the current density on the Vickers hardness of 3YSZ/Al_2_O_3_ composites. The inset shows the indentation patterns on the surfaces of FS-102.

Figure 5 shows the average Vickers hardness of the 3YSZ/Al_2_O_3_ composites as a function of current densities. As mentioned earlier, the density increased as the current density increased, which improved the mechanical property of the samples. Therefore, the average hardness also increased with the increase of the current density. For a current density from 51 mA/mm^2^ to 76 mA/mm^2^, the slope of the increase in hardness was smaller, while the increase in hardness was faster for a current density from 76 mA/mm^2^ to 102 mA/mm^2^. In particular, the hardness of the FS-102 sample was 11.3 GPa, which was comparable to that of the CS sample (11.4 GPa). The inset shows that no cracks were observed at the indentation, indicating the good fracture toughness of the flash-sintered 3YSZ/Al_2_O_3_ composite [16].

## 4. Conclusions

The 3YSZ/Al_2_O_3_ composites were prepared by flash sintering at a low furnace temperature (700 °C). The current density presented a significant effect on the relative density and grain size of the samples. The grain sizes, relative densities, and Vickers hardness of the samples increased with the increase of the current density. A high relative density of 94.2% and a maximum Vickers hardness of 11.3 GPa were obtained when the current density was 102 mA/mm^2^, which was comparable to those of the sample prepared by conventional sintering. The results of the sample temperature estimation suggest that Joule heating and defects generation are the main causes of rapid densification in flash sintering. Due to the high local temperature of the sample, the molten zone presented eutectic structures, including colony, regular, and irregular eutectic structures. Thus, it is necessary to consider the possibility that the local liquid phase generated during flash sintering is due to grain boundary overheating. This work paves a new way for optimizing the microstructure and properties of multiphase oxide ceramics during fabrication by flash sintering.

## Figures and Tables

**Table 1:** The relative density, average grain size, and estimated sample temperature of 3YSZ/Al_2_O_3_ composites under different sintering conditions.

Sample	Average Grainsize (μm)		Relative Density (%)	Sample Temperature (°C)
	ZrO_2_	Al_2_O_3_		
FS-51	0.18	0.22	83.6	1130
FS-64	0.23	0.27	84.1	1221
FS-76	0.29	0.32	85.5	1297
FS-89	0.31	0.41	92.7	1391
FS-102	0.33	0.47	94.2	1467
CS	0.30	0.36	93.2	1400

## Data Availability

Data available on request due to privacy restrictions. The data pre-sented in this study are available on request from the corresponding author.
